# Comparing the Effects of Antenatal Betamethasone on Doppler Velocimetry Between Intrauterine Growth Restriction With and Without Preeclampsia

**DOI:** 10.5539/gjhs.v7n2p344

**Published:** 2015-01-12

**Authors:** Kobra Shojaei, Nooshin Mohammadi

**Affiliations:** 1 Fertility Infertility and Perinatology Research Center, Department of obstetrics & gynecology, Ahvaz Jundishapur University of Medical Sciences (AJUMS), Ahvaz, Iran; 2 Department of Obstetrics & Gynecology, Kermanshah University of Medical Sciences, Kermanshah, Iran

**Keywords:** preeclampsia, betamethasone, corticosteroids, intrauterin growth restriction, followup studies, Doppler Ultrasonography

## Abstract

Evaluation of the effects of betamethasone on patients with intrauterine growth restriction in couple with preeclampsia is not well studied. This study was designed to assess and compare the changes of Doppler flow in maternal, fetal and placental arteries in singleton pregnancies complicated by IUGR and preeclampsia which are at 24-34 weeks of gestation after betamethasone therapy. This prospective, longitudinal and multicenter study was conducted in 2013 on the 40 singleton pregnant women with IUGR fetuses and concerned over maternal or fetal well-being. Three Doppler measurements including absolutely before betamethasone, one day after betamethasone and 5 days after betamethasone administration were performed. Flow velocity waveforms were obtained from uterine arteries (UA), Umbilical (UM), and middle cerebral artery (MCA). The Systolic/Diastolic ratio (S/D), Resistance Index (RI), and Pulsatility Index (PI) were determined for waveforms. Comparison of baseline mean scores between IUGR with and without preeclampsia showed no statistically significant differences. The mean scores of UA, MCA-UM-RI, UM-S/D, UM-PI, and UM-RI did not differ statistically significant between three time points when compared trend between negative and positive preeclampsia subjects. However, UM-RI had close P value to the margin of statistical significance (P value = 0.055). In other words, in our study, UM-RI had a clear tendency to be significance. We can conclude that preeclampsia alone could not be major prognostic factor in pregnancies with IUGR. While, other prognostic factors such as gestational age, fetal weight, and fetal vascular Doppler flow may are more important for decision making about termination of preeclampsia.

## 1. Introduction

Preeclampsia usually presents as high blood pressure and proteinuria after 20 weeks of gestation (National Institiutes of Health, 2000; Helewa et al., 1997; ACOG Committee on Obstetric Practice, 2002). Intrauterine Growth Restriction (IUGR) is defined as the failure of the fetus to attain its expected weight. The most common definition for IUGR is the estimated fetal weight less than 10th percentile for gestational age (Callen, 2008). Preeclampsia is often associated with IUGR and oligohydramnios due to chronic placental hypoperfusion. In the presence of IUGR the preeclampsia is termed as severe preeclampsia. IUGR with early onset preeclampsia (severe preeclampsia) can more reduce blood flow to the placenta compared with normal pregnancies (23% versus 12%) (Phyllis & Sibai, 2013).

Abnormal implantation along with the syndrome of preeclampsia and IUGR is the major risk factor for maternal and perinatal morbidity and mortality. Early onset preeclampsia contributes greatly to IUGR, abnormal umbilical and uterine artery blood flow, and adverse outcomes for both mother and fetus. It is suggested that vascular disorders may lead to abnormal implantation and consequently vascular diseases in the mother. The evaluation of arterial stiffness by Pulsed Wave Analysis (PWA) showed more arterial stiffness in women with early onset preeclampsia or with IUGR but normal blood pressure compared with women with late onset preeclampsia or healthy women (Yinon et al., 2010). In addition, corticosteroid therapy is effective in reducing the incidence of respiratory distress and other adverse consequences of preterm delivery including intraventricular hemorrhage, necrotizing enterocolitis, and fetal death (Crowther & Harding, 2007). As NICE (National Institute for Health and Clinical Excellence) suggests 2 doses of 12 mg muscular betamethasone for intrauterine growth restriction at 24 to 34 weeks of gestation and as well as with probability of delivery during one week after this time (NICE, 2010).

Doppler ultrasonography is well-known as a non-invasive procedure for evaluating surveillance of the fetus and as well as vascular blood flow velocity such as umbilical artery, umbilical vein, aorta, middle cerebral artery. For example, a meta-analysis of randomized controlled trials of the high risk pregnancies showed that umbilical artery Doppler investigation is an effective test for reducing morbidity and mortality of fetus (Maulik, 2013). Comparing the effects of betamethasone between intrauterine growth restriction with preeclampsia and without preeclampsia is not well evaluated.

This study was designed to assess and compare changes of Doppler flow in maternal, fetal and placental arteries in singleton pregnancies complicated by IUGR and preeclampsia which are at 24-34 weeks of gestation after betamethasone therapy.

## 2. Materials and Methods

### 2.1 Study Design and Population

This prospective, longitudinal and multicenter study was conducted in 2013 on the 40 singleton pregnant women with IUGR fetuses and concerned over maternal or fetal well-being. Women of our study were complicated with intrauterine growth-restriction (estimated fetal weight below the 10^th^ percentile for gestational age). In other words, they had high probability of preterm delivery and termination of pregnancy.

### 2.2 Place of the Study

Patients were referred to three university affiliated and tertiary prenatal care clinics for prenatal care including Imam Khomeini Hospital, Women Hospital, and Shariati Hospital, Tehran, Iran.

### 2.3 Inclusion Criteria

All the women received betamethasone (BETAMETHASONE LA DP 1ML AMP) for fetal lung maturation and fulfilled the following inclusion criteria: (1) gestational age at 24 to 34 weeks (based on Last Menstrual Period) (2) singleton pregnancies in which no fetal anomalies had been detected by ultrasonography, (3) the subsequent administration of a course of betamethasone, and (4) fetuses with confirmed IUGR.

### 2.4 Exclusion Criteria

Exclusion criteria were (1) structural, congenital anomalies, or chromosomal disorders (2) patients who could not complete the study due to Intra-Uterine Fetal Death (IUFD) or need to emergency terminate the pregnancy and (3) starting betamethasone therapy course before enrolling in the study.

### 2.5 Ethical Considerations

Participants’ informed consent was gained; voluntary participation and confidentiality were guaranteed. We had not any missing data. The study was approved by the Ethics Committee of the department of Obstetrics and Gynecology (Tehran University of Medical Sciences).

### 2.6 Procedure

Three Doppler measurements absolutely before betamethasone, one day after betamethasone and 5 days after betamethasone administration were performed. For all cases two doses of 12 mg betamethasone given intramuscularly repeated at 24-hour apart. All Doppler studies were performed by ultrasound examinations. Doppler Ultrasonography was performed using A Acuson Antares Ultrasound System (Siemens AG Co., Germany).

### 2.7 Data Collection

Characteristics data included maternal age, gestational age at diagnosis, BMI (Body Mass Index), maternal blood pressure (BP), maternal diabetes, preeclampsia and parity. Preeclampsia was defined as systolic BP>= 140 mmhg or diastolic BP >= 90 mmhg and proteinuria >= 300 mg/day after 20-week of gestation. Flow velocity waveforms were obtained from the left and right maternal uterine arteries (UA), fetal umbilical artery (UmA), fetal middle cerebral artery (MCA), and MCA-RI/UmA-RI ratios. All recording were taken during fetal inactivity to ensure steady signals in the venous channel. The systolic/diastolic ratio (S/D), resistance index (RI), and pulsatility index (PI) were determined for waveforms.

### 2.8 Data Analysis

The analysis was carried out with SPSS software program version 17. Continuous variables were described with mean ± standard deviation, and qualitative variables were expressed as percentage value. Analysis of variance for repeated measures was used to study the Doppler velocity waveform patterns of UA, UmA, MCA, and MCA-RI/UmA-RI longitudinally over three time points. Independent t-test was used to compare baseline mean of blood flow velocity between preeclampsia and non preeclampsia groups. Significance level (P-Value) of 0.05 was deemed to indicate the statistically significant difference for all tests.

## 3. Findings

Among 40 singleton pregnant women with IUGR, 16 (40%) had preeclampsia. For all patients, mean ± SD score for maternal age was 29.8 ± 7.7 years old, gestational age was 29.7 ± 2.8 weeks, and BMI was 26.2 ± 3.8 kg.

**Figure 1:**
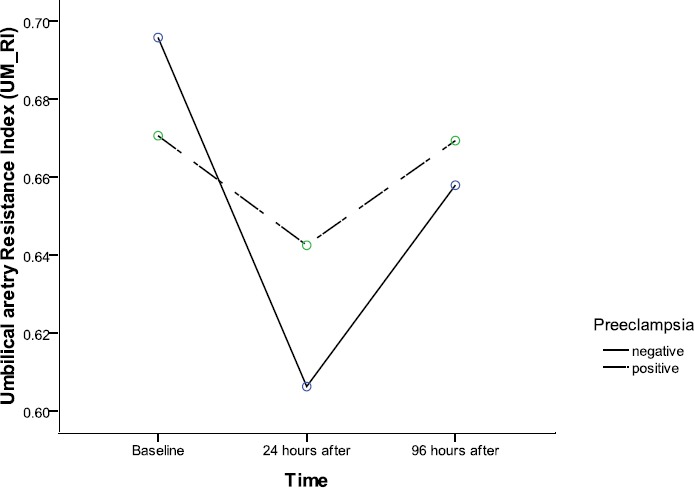
Comparing the effects of maternal betamethasone therapy on umbilical artery pulsatility index between IUGR with and without preeclampsia

Independent t-test results for patients’ characteristics between positive and negative preeclampsia groups are given in Table 1. As can be seen, averages of mothers’ age, mothers’ BMI, gestational age, and fetal weight for the negative and positive preeclampsia in the independent t-test were not significantly different. While, systolic BP (P value <0.0001) and diastolic BP (P value <0.0001) which are the criteria for distinguishing positive from negative preeclampsia were statistically significant. Table 2 represents comparison of baseline the mean scores between IUGR with and without preeclampsia. There were no statistically significant differences between two groups at baseline time. The repeated measure ANOVA determined that the mean scores of UA, MCA-UM-RI, UM-S/D, UM-PI, and UM-RI did not differ statistically significant between three time points when compared trend between negative and positive preeclampsia subjects (Table 3). However, UM_RI had close P value to the margin of statistical significance (P value = 0.055). In other words, in our study, UM_RI had a clear tendency to significance (Figure 1, Table 3).

**Table 1:** Independent T-test results for patients’ characteristics between IUGR with and without preeclampsia (n= 40)

Characteristics	Preeclampsia		P value

	Positive (n= 16) (mean ± SD)	Negative (n = 24) (mean ± SD)	
Mother’s Age (year)	27.9 ± 8.4	31.0 ± 7.0	0.2
Mother’s BMI (kg/m^2^)	25.8 ± 4.2	26.5 ± 3.5	0.5
Systolic BP (mmhg)	152.19 ± 7.739	109.6 ± 9.1	<0.0001*
Diastolic BP (mmhg)	96.25 ± 5.627	67.1 ± 7.5	<0.0001*
Pregnancy Age (week)	29.1 ± 3.0	29. 1± 2.6	0.6
Weight of Fetus (gr)	919.1 ± 346.8	967.6 ± 324.9	0.6

**Table 2:** Results of independent t-test between baseline mean of blood velocity in intrauterine growth restriction with preeclampsia and without preeclampsia

Arteries	Preeclampsia at baseline		

	Negative (n= 24)	Positive (n=16)	P value
UA-PI	.95 (.4)	.98 (.3)	0.08*
MCA-RI	.75 (.1)	.75 (.1)	0.9
UM-RI	.69 (.1)	.69 (.1)	0.8
UM-PI	1.17 (.2)	1.5 (.2)	0.3
UM-SD	3.5 (.8)	3.6 (.9)	0.7
MCA-UM-RI	1.11 (.3)	1.1 (.3)	0.7

†Data are reported as mean (standard deviation).

* Statistically significant (at level of 0.05).

UA= Uterine Artery; UM = UMbilical artery; MCA =Middle Cerebral Artery. PI = Pulsatility Index; S/D= Systolic/Diastolic ratio; RI= Resistance Index;

**Table 3:** Results of ANOVA-repeated measures for Doppler velocimetry at three time points in women with IUGR administrated by betamethasone therapy

Arteries	Preeclampsia								

	Positive				Negative				^α3^P value
	Baseline	1-day after	5-day after	^α1^P value	Baseline	1-day after	5-day after	^α2^P value	
		
UA	†.98 (.3)	.93 (.3)	.93 (.3)	<.001	1 (.4)	.9(.4)	.9(.4)	*<.001	0.2
MCA-RI	.75 (.1)	.8 (.1)	.78 (.1)	<.001	.8 (.1)	.77 (.1)	.78 (.1)	<.001	0.6
UM-RI	.67 (.1)	.64 (.1)	.67 (.1)	<.001	.69 (.1)	.61 (.1)	.65 (.1)	<.001	0.055
UM-PI	1.2 (.2)	1.1 (.1)	1.1 (.2)	<.001	1.2 (.1)	.99 (.1)	1.1 (.2)	<.001	0.1
UM-SD	3.6 (.9)	3.3 (.9)	3.3 (.9)	<.001	3.6 (.8)	3.03 (.6)	3.3 (.7)	<.001	0.3
MCA-UM-RI	1.14 (.2)	1.2 (.2)	1.2 (.3)	<.001	1.1 (.3)	1.3 (.3)	1.2 (.3)	<.001	0.1

† Data are reported as mean (standard deviation);

* Statistically significant (at level of 0.05);

α1: P value of changes within 5-day follow up in IUGR with preeclampsia group;

α2: P value of changes within 5-day follow up in IUGR without preeclampsia group;

α1: P value of differences between IUGR with and without preeclampsia groups within 5-day follow up;

PI = Pulsatility Index; S/D= Systolic/Diastolic ratio; RI= Resistance Index;

UA= Uterine Artery; UM = UMbilical artery; MCA =Middle Cerebral Artery.

## 4. Discussion

Women with intrauterine growth restriction and early-onset preeclampsia (n=16) and intrauterine growth restriction without preeclampsia (n=24) were studied at 24 to 34 weeks of gestation. Measurements at three time points were conducted to study and compare effects of betamtethasone usage on vascular dysfunction in both groups. We found that betamethasone therapy had significant effects on UA-PI, UM-PI, UM-RI, UM-S/D, MCA-RI, and MCA-UM-RI over 5-day follow up time period in each group (P value for each artery in each group was <0.0001) (Table 3).

In some studies, no sever adverse effects after betamethasone administration have been reported during second half of pregnancy. However, some other investigations found adverse neonatal effects such as decreased fetal movement, respiratory activation, and beat to beat variability after betamethasone usage (Mulder, Derks, Zonneveld, Bruinse, & Visser, 1994; Derks, Mulder, & Visser, 1995). These side effects are transient and their permanent duration is only 4 days after betamethasone initiation. Although, there is some evidence that shows association between repeating course of betamethasone and higher risk of IUGR (Crowther & Harding, 2007). But in our study, we did not observed any adverse effects during 5 days follow up.

Betamethasone administration improves end diastolic blood flow of umbilical artery in fetuses with IUGR or placental insufficiency symptoms (Wallace & Baker, 1999; Edwards, Baker, & Wallace, 2002). There is insufficient data about impact of betamethasone usage on the fetal ductus venosus blood flow. In early onset preeclampsia, abnormal implantation can increase the impedance of the uterine artery flow which can be represented by increased pulsatility index. Elevated pulsatility index sometimes is associated with uterine artery notching in Doppler sonography. In addition, increased placental vascular resistance is associated with increasing indices of umbilical artery Doppler (Phyllis & Sibai, 2013).

Fetal ductus venosus blood flow is valuable in taking an accurate decision about timing of pregnancy termination in the intrauterine growth restriction syndrome (Hecher et al., 2001; Ferrazzi, 2002; Romero, Kalache, & Kadar, 2002). Our findings showed decreased level of UM-PI during five days after betamethasone initiation (Table 2). But this reduction was transient. Actually at the end of five-day follow up time, its value returned to baseline level. Pattern of decreasing between IUGR with preeclampsia and without preeclampsia was similar (P value = 0.1).

Evidences about fetuses at risk of preterm delivery or placental insufficiency showed contrast results. It had been shown that betamethasone does not change umbilical artery pulsatility index in perinatal period (Cohlen et al., 1996; Deren et al., 2001).

Another studies reported decreased MCA-PI after maternal betamethasone therapy (Chitrit et al., 2000; Piazze et al., 2001). In a study done by Piazza and et al. (2011), they showed significant but transient changes of betamethasone usage on Doppler of maternal-fetal blood flow (Piazze et al., 2001).

In a study by Yinon and et al., they compared vascular dysfunction between four groups including early-onset preeclampsia, late-onset preeclampsia, and intrauterine growth restriction without preeclampsia, and prior normal pregnancy at postpartum months. They concluded that risk of future vascular disease was higher only in women with a history of early-onset preeclampsia or intrauterine growth restriction without preeclampsia (Yinon et al., 2010). Their findings support our results regarding similarity of vascular function between IUGR with preeclampsia and without preeclampsia groups. These similarities may indicate that pathogenesis of IUGR results from placental-fetal vascular dysfunctions. Several other studies showed that IUGR and preeclampsia have potential of endothelial dysfunction which leads to inappropriate placental implantation (Ness & Sibai, 2006; Roberts, & Catov, 2008; Yinon et al., 2010). In another study done by Villar and et al, suggested that IUGR seems to have similar etiology with preeclampsia but biologically is different (Villar et al., 2006). In a study conducted in the Philadelphia has been reported that preeclampsia plays direct role in IUGR incidence in which severe preeclampsia is associated with higher risk of IUGR in compared with mild preeclampsia (Ness & Sibai 2006). We found that prevalence of preeclampsia in IUGR was 16 cases out of 40 IUGR fetuses (40%) which is high proportion.

Finally, we found that betamethasone therapy had similar effects on maternal, placental and fetal arteries blood flow velocity between intrauterine growth restriction with and without preeclampsia within 5-day follow-up based on Doppler ultrasonography studies.

## 5. Conclusion

We can conclude that preeclampsia alone could not be major prognostic factor in pregnancies with IUGR. While other prognostic factors such as gestational age, fetal weight, and fetal vascular Doppler flow may are more important for decision making about termination of preeclampsia. As our study showed, the response of IUGR with and without preeclampsia to betamethasone therapy was the same within 5 days follow up. Therefore, it is more probable that placental vascular dysfunction may lead to intrauterine growth restriction. At the end, we suggest that perinatologists should be more careful in interpreting Doppler ultrasonography reports of IUGR fetuses. Also, it is better to take more attention to other prognostic factors for termination of pregnancy.
